# Production of Biodegradable Film Containing Wheat Leaf Fiber and Protein Isolate, Reinforced With Encapsulated Silver Oxide Nanocomposite, as a Color Indicator for Monitoring the Spoilage of Minced Chicken Meat

**DOI:** 10.1002/fsn3.71805

**Published:** 2026-07-29

**Authors:** Mandana Yekta, Sedigheh Yazdanpanah, Mohammad Hosein Marhamatizadeh, Dornosh Jafarpour

**Affiliations:** ^1^ Department of Food Hygiene, Kaz.C. Islamic Azad University Kazerun Iran; ^2^ Department of Food Science and Technology, Kaz.C. Islamic Azad University Kazerun Iran; ^3^ Department of Food Science and Technology, Fa.C. Islamic Azad University Fasa Iran

**Keywords:** chicken meat, nanocomposite, protein isolate, wheat leaf fiber

## Abstract

Bio‐based films have garnered significant attention in the food packaging industry due to their eco‐friendliness and multifunctional properties. This study aimed to evaluate the effects of silver oxide nanocomposites and encapsulation processes on the physical, chemical, thermal, and microbiological properties of bio‐based films. Three types of films were prepared and analyzed, including a base film (containing protein and plant fiber), a film with silver oxide nanocomposites, and an encapsulated film with silver oxide nanocomposites. FTIR, TGA, DSC, SEM, and microbiological tests were conducted to evaluate the structural, thermal, and antimicrobial properties of the films. Statistical analysis was performed using SPSS at a significance level of *α* = 0.05. The *t*‐Test was used for microbiological tests, while Duncan's test was applied for other data. Results showed that the addition of silver oxide nanocomposites, particularly in the encapsulated film, significantly enhanced antibacterial performance, thermal stability, and mechanical properties. The encapsulated film effectively reduced the growth of pathogenic bacteria such as *Staphylococcus aureus* and *Escherichia coli* and prevented Salmonella growth. Additionally, it minimized lipid oxidation and maintained the natural color of chicken meat during storage. This study demonstrated that the incorporation of silver oxide nanocomposites and encapsulation technology can contribute to the development of active and intelligent packaging solutions to extend food shelf life.

## Graphical abstract

Encapsulated silver oxide nanocomposite‐reinforced protein–fiber biodegradable films exhibited enhanced antioxidant, antimicrobial, and mechanical properties. The encapsulated film effectively inhibited *Staphylococcus aureus* , *Escherichia coli* , and prevented Salmonella growth, while preserving the chemical quality and color stability of minced chicken meat during 15 days of storage, demonstrating its potential as an active and intelligent food packaging system.

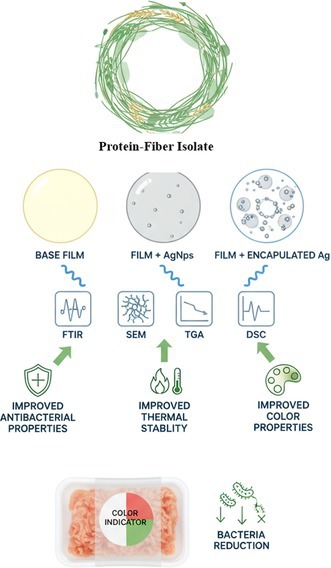

## 1 Introduction

The growing global concern over the environmental impacts caused by the extensive use of non‐degradable plastics has shifted the focus of the scientific community toward the development of biodegradable materials (de Sadeleer and Woodhouse 2024; Haider et al. 2019). Traditional plastics, due to their long‐lasting persistence in the environment and widespread accumulation in ecosystems, have become one of the most significant environmental challenges of the 21st century. This issue is particularly critical in the food packaging industry, where sustainable and eco‐friendly materials are urgently needed (El‐Sayed and Youssef 2023; Hussain et al. 2024). The production of biodegradable films as an alternative to conventional plastics is an innovative solution that has gained attention over the past decade (Moshood et al. 2022). Biodegradable materials are primarily derived from renewable agricultural or biological resources and are highly valued for their unique properties, such as environmental compatibility, high biodegradability, and suitable mechanical characteristics (Mukherjee et al. 2023). Among these, wheat plant leaves, as one of the agricultural by‐products with high added value, have shown great potential due to their natural fiber content and isolated proteins. These materials can provide acceptable mechanical performance and serve as a basis for developing sustainable and eco‐friendly films (Liu et al. 2023; Plakantonaki et al. 2023). In addition to the use of bio‐based materials, incorporating these films with nanoparticles and nanocomposites can enhance their functional properties. In this regard, silver oxide nanocomposites have received particular attention due to their strong antibacterial properties, high reactivity, and environmental compatibility (Cobos et al. 2020; Danish et al. 2022). These nanocomposites, when encapsulated within bio‐matrices, can reinforce the antibacterial performance of biodegradable films and extend the shelf life of food products. Furthermore, due to their color‐changing capabilities under specific conditions, these nanocomposites can act as colorimetric indicators in intelligent packaging (Casalini et al. 2023; Kishore et al. 2023; Singh and Kumar 2023).

In this study, wheat plant leaves were selected as a raw material because they are an abundant agricultural by‐product rich in natural fibers and proteins, offering an eco‐friendly and cost‐effective alternative to conventional biopolymers. Utilizing wheat leaves contributes to waste valorization and aligns with the principles of circular bioeconomy by converting underutilized residues into high‐value materials (Liu et al. 2023; Plakantonaki et al. 2023). Silver oxide nanocomposites (Ag_2_O) were incorporated due to their strong antimicrobial properties, high thermal stability, and compatibility with biodegradable matrices, which make them ideal for active packaging applications (Danish et al. 2022; Kraśniewska et al. 2020). Glycerol was employed as a natural, non‐toxic plasticizer to enhance film flexibility and reduce brittleness without compromising biodegradability, which has been widely reported as effective in biopolymer‐based films (Vieira et al. 2022; Cofelice et al. 2019). The combination of these components allows the development of a sustainable, functional, and antimicrobial biofilm suitable for intelligent food packaging systems.

The application of colorimetric indicators in food packaging, especially for protein‐based products such as minced chicken meat, is a practical innovation. Food spoilage, typically caused by microbial growth and chemical changes in its components, not only reduces the quality and safety of food but also poses risks to consumer health. Colorimetric indicators visually display the chemical changes associated with spoilage, allowing consumers to quickly and reliably evaluate the quality of food products (Almasi et al. 2022; Chiu and Yang 2024; Luo et al. 2022). Although previous studies have demonstrated that the use of biodegradable films and colorimetric indicators in intelligent packaging can reduce food waste and improve food safety, combining biodegradability, antibacterial properties, and colorimetric indication in a single packaging film remains a scientific and technological challenge (Amin et al. 2022; Moustafa et al. 2025; Yu et al. 2023). In this study, we aim to develop a biodegradable film based on fiber and isolated protein from wheat plant leaves, reinforced with encapsulated silver oxide nanocomposites, and evaluate its performance as a colorimetric indicator for monitoring the spoilage of minced chicken meat. This research utilizes natural resources, advanced technologies, and a sustainable approach to contribute to the development of intelligent and eco‐friendly packaging solutions.

## 2 Materials and Methods

Fresh wheat leaves were obtained from local agricultural sources. Silver oxide nanocomposites were synthesized in‐house using a chemical reduction method with silver nitrate (AgNO_3_, Sigma‐Aldrich) and sodium hydroxide (NaOH, Merck). Glycerol (Merck) was used as a plasticizer in varying concentrations (10%–30% w/w) relative to the dry weight of the protein isolate.

### 2.1 Extraction of Protein‐Fiber Isolate From Wheat Leaves and Encapsulation of Protein‐Fiber Isolate With Silver Oxide

Wheat stem powder was combined with a 1% (w/v) sodium hydroxide solution at a 1:40 ratio and stirred on a magnetic stirrer. An ultrasonic probe (Branson 2510, USA) was submerged to a depth of 20 mL in the solution, and extraction was conducted at 40°C for 60 min with 400 watts of ultrasonic power. The resulting solution was centrifuged using an Eppendorf 5804 centrifuge (Germany) at 7000 rpm for 15 min. Wheat leaves were washed with cold water to remove impurities, air‐dried, and ground into a fine powder using a high‐speed grinder (Moulinex AR1100, France), and then sieved through a 60‐mesh sieve. The powder was defatted with hexane (1:5 ratio) for 2 h and filtered, then stored at 4°C until protein extraction. For protein extraction, ultrasonic waves from a probe‐type device (Hielscher UP200Ht, Germany) with 100 watts of power were applied to the mixture of ground samples and solvent for 2 min at 20°C. The mixture was refrigerated at 4°C for 24 h to precipitate non‐protein compounds and improve yield. The phases were separated using a Sigma 3‐18KS centrifuge (Germany) at 7516 rpm for 10 min. The pH of the upper phase was then adjusted to the isoelectric point using 2 N hydrochloric acid. The mixture was centrifuged again, the upper liquid was discarded, and the protein precipitate was dried using a freeze dryer, model Labconco FreeZone 6 L (USA), for 48 h. For encapsulation, 5 g of extracted fiber powder, protein isolate, and silver oxide were combined. A 0.1% (w/v) sterile solution was prepared and mixed with 20 mL of 2% sodium alginate dissolved in 5 mL of sterile peptone water (sterilized at 121°C for 15 min). The suspension was injected into a 0.1% sterile calcium chloride solution using a syringe. The capsules were hardened in the solution at 5°C for 30 min, then rinsed and stored in sterile peptone water until use (Chen et al. 2023; Singh et al. 2023; Tahir et al. 2024). The characteristics and preparation methods of the free and encapsulated combinations are summarized in Table 1.

**TABLE 1:** Characteristics of protein, fiber, and silver oxide (free and encapsulated combination).

Type of combination	Preparation process
Free combination (protein, fiber, and silver oxide)	Protein extracted from wheat leaves (precipitation at isoelectric point and freeze‐drying), fiber isolated (defatted with hexane and ground), and pure silver oxide powder prepared
Encapsulated combination (protein‐fiber‐silver oxide)	Mixed raw materials with 2% sodium alginate solution, injected into 0.1% calcium chloride solution, hardened at 5°C, washed, and stored in sterile solution

### 2.2 Scanning Electron Microscopy

Scanning electron microscopy (SEM) images of the surface and cross‐section of the films were taken using a TESCAN VEGA3 microscope (Czech Republic). Surface samples were mounted on aluminum stubs with double‐sided tape. Cross‐sectional samples were fractured in liquid nitrogen and mounted similarly. The stubs were gold‐coated for 5 min using a Quorum Q150R ES coater (UK). Imaging was performed at various magnifications to analyze the structure (Tahir et al. 2024).

### 2.3 Antioxidant Activity

The antioxidant capacity was measured using the DPPH free radical scavenging method. Initially, the DPPH solution was prepared, and samples were diluted at a ratio of 18:1. Subsequently, 25 μL of the sample was mixed with 100 μL of the DPPH solution and incubated in darkness at room temperature for 20 min. The absorbance of the mixture was then measured at a wavelength of 517 nm using a spectrophotometer, allowing for the evaluation of the antioxidant activity of the samples (Avci et al. 2024).

### 2.4 Antimicrobial Activity Assessment

The bacterial suspension was prepared to match a turbidity of 0.5 McFarland standard, equivalent to approximately 1 × 10^8^ CFU/mL. The turbidity was adjusted using physiological saline as necessary. Using a sterile swab, the bacterial suspension was evenly spread on Mueller‐Hinton agar plates. The inoculation was performed using a streaking method in three different directions to ensure even distribution. Antimicrobial disks were placed on the agar surface using sterile forceps, ensuring that they were evenly spaced. The plates were incubated at 37°C for 24 h. After incubation, the diameter of the inhibition zones around each disk was measured. The size of the inhibition zone indicates the susceptibility of the specific bacteria to the antimicrobial agent (Alamier et al. 2023).

### 2.5 Film Production

The extracted protein, fiber, and encapsulated silver oxide nanocomposites were dissolved in 100 mL of distilled water (Table 2). The concentrations and proportions of each component were determined based on preliminary optimization trials and previous studies. Specifically, 5 g of extracted protein and fiber per 100 mL of distilled water provided optimal film‐forming ability and homogeneous texture, while preventing aggregation. Silver oxide nanocomposites were added at a concentration ensuring effective antimicrobial activity without compromising film transparency or flexibility (del Rosario Herrera‐Rivera et al. 2024; Vieira et al. 2022). Glycerol was incorporated at 45% (w/w, based on the dry weight of protein) as it enhanced elasticity and reduced brittleness, consistent with earlier findings (Akachat et al. 2025; Cofelice et al. 2019). These ratios achieved a balance between mechanical strength, antimicrobial performance, and optical properties. The mixture was stirred using a magnetic stirrer at 30°C for 1 h. The prepared solution was exposed to ultrasonic waves using a probe at a duty cycle of 120 s ON and 15 s OFF at 50% amplitude for 15 min, and 5 g of the sample were added and stirred for an additional hour. The solution was heated in a water bath at 90°C for 30 min. The solution was allowed to cool for 20 min to remove air bubbles. The film‐forming solution (100mL) was poured into Teflon containers with a diameter of 16 cm. The solution was dried at 25°C for 24 h to achieve a uniform thickness. The films were stored in a desiccator at 23°C with a relative humidity of 50% (±5%) until testing (García et al. 2024; Tafa and Engida 2022).

**TABLE 2:** Composition and preparation details of film samples.

Sample	Ingredients	Amount of ingredients per 100 mL distilled water	Glycerol percentage based on protein dry weight	Production conditions
Control film	Protein, extracted fiber	5 g protein, fiber, and nanocomposites	45% glycerol	—
Non‐encapsulated film	Protein—Extracted fiber—Silver oxide nanocomposites—Glycerol	5 g protein, fiber, and nanocomposites	45% based on protein dry weight	Dissolved at 30°C for 1 h‐ Ultrasonicated at 50% amplitude (120 s ON, 15 s OFF)—Dried for 24 h
Encapsulated film	Encapsulated protein—Extracted fiber—silver oxide nanocomposites	5 g protein, fiber, and nanocomposites	45% based on protein dry weight	Dissolved at 30°C for 1 h‐ Ultrasonicated at 50% amplitude (120 s ON, 15 s OFF)—Dried for 24 h

*Note:* Control Film: Film containing 5 g of protein, extracted fiber from wheat leaves per 100 mL of solution. Non‐Encapsulated Film: Film containing 5 g of protein‐extracted fiber and silver oxide nanocomposites per 100 mL of solution. Encapsulated Film: Encapsulated film containing 5 g of protein‐extracted fiber and silver oxide nanocomposites per 100 mL of solution.

### 2.6 Evaluation and Measurement Methods of Film Properties

The thickness of films was measured at 10 different points using a digital micrometer, model Mitutoyo 293‐340‐30 (Japan), with an accuracy of 0.001 mm, and the average values were used to determine tensile strength and water vapor permeability. Moisture content was assessed by cutting samples into 2 cm^2^ squares, weighing them using a digital balance (Kern ABT 120‐5DM, Germany), with an accuracy of 0.001 g, drying them in an oven (Memmert UN110, Germany), at 100°C for 24 h, and calculating the percentage moisture content based on initial and final weights. Water solubility was determined by cutting 4 cm^2^ samples, drying them in an oven (Carbolite GPC12, UK) at 105°C for 24 h, immersing them in distilled water for 1 to 4 min, and weighing them before and after drying to calculate solubility. Tensile tests were performed on 10 × 54 cm samples, with a jaw distance of 50 mm and a jaw speed of 50 mm/min, using a fabric testing machine (Instron 5565, USA), with at least three repetitions. Tensile strength (TS) and elongation at break were measured at the point of failure (Abdulwadood et al. 2024; Javan et al. 2024).

### 2.7 Hydrophobicity Assessment

The hydrophobicity of the film was determined by measuring the contact angle of a water droplet on the film surface. The contact angle was measured using a setup equipped with a camera and an adjustable tilt base. A 5 μL water droplet was randomly placed on five points of the film surface using a micropipette. The average of these measurements was reported as the static contact angle (Caruso et al. 2023).

### 2.8 Turbidity Measurement of Films

To assess light transmission and turbidity, film samples were placed in spectrophotometric cells of the Shimadzu UV‐1800; Japan. Scans were performed at wavelengths of 200 to 600 nm to measure light transmission. The turbidity of the films was calculated using the following relationship:

Turbidity=Absorbanceat600nm\Film Thickness

This approach allowed for a comprehensive evaluation of the optical properties of the films.

### 2.9 FRAP

The reducing power of each sample was evaluated based on the reduction of potassium ferricyanide and the color change from yellow to green or blue. A sample of 0.03 g was mixed with 5 mL of 0.1% potassium ferricyanide and 5 mL of sodium phosphate buffer (pH 6.5). The mixture was incubated in a water bath at 50°C for 20 min. Following incubation, 2.5 mL of 10% trichloroacetic acid solution was added to the tubes and centrifuged at 1650 g for 20 min at room temperature. A 0.5 mL aliquot of the supernatant was mixed with 0.5 mL of ferric chloride and 1.5 mL of distilled water. The absorbance was measured at a wavelength of 700 nm using a Shimadzu UV‐1700 spectrophotometer. An increase in absorbance indicated an increase in the reducing power of the sample, which reflects its antioxidant activity (Adjimani and Asare 2015).

### 2.10 Surface Color Measurement

The surface color of the films was measured using a colorimeter. Samples were placed on a standard white tile, and the color parameters were determined, specifically the Δ*E* value for yellow/blue and the parameters a* (red/green) and b*. The relationship for calculating the color difference (Δ*E*) was given by the following formula:

$$∆E=\sqrt{{\left({\left(2^{-L}\right)}^{2}⇍\left(a^{2^{-a}}\right)\right)}^{2}+{\left(u_{2}-b_{1}\right)}^{2}}$$

### 2.11 Color Changes in Indicator Films

To evaluate the color changes of the indicator films, solutions with different pH values (ranging from 2 to 13) were prepared using NaOH and HCl. Initially, 2 cm × 2 cm pieces of the indicator films were cut and exposed to 10 μL of the prepared solutions for 2 min. Subsequently, the films were placed in various pH solutions (2–13). Photographs of the samples were then taken under identical conditions to document the color changes (Ezati and Rhim 2021).

### 2.12 Fourier‐Transform Infrared Spectroscopy (FTIR)

A portion of the film sample was prepared and placed inside a Fourier‐Transform Infrared Spectrometer (PerkinElmer Frontier, USA) for analysis. The spectral data were collected within the wavenumber range of 4000 to 400 cm^−1^ with a resolution of 4 cm^−1^. This analysis aimed to identify the functional groups present in the film based on the characteristic absorption peaks observed in the infrared spectrum (Chen et al. 2015).

### 2.13 Thermogravimetric Analysis/Differential Scanning Calorimetry (TGA/DSC)

TGA and DSC analyses were performed on the film samples using a thermogravimetric analyzer (Mettler Toledo TGA/DSC 3+, Switzerland). The analysis was conducted in the temperature range of 0°C–500°C, with a heating rate of 10°C per minute, and lasted for 45 min. Helium gas was used as the analysis atmosphere to ensure accurate thermal decomposition and phase transition data (Shen et al. 2015).

### 2.14 Microstructural Analysis of the Produced Film (SEM)

Scanning electron microscopy (SEM) images of the surface and cross‐section of the films were captured using a TESCAN VEGA3 SEM (TESCAN, Czech Republic). For surface imaging, the samples were mounted onto aluminum stubs using double‐sided adhesive tape. For cross‐sectional imaging, the samples were first fractured in liquid nitrogen, and the fractured surface was then mounted onto aluminum stubs with adhesive tape. The stubs were gold‐coated for 5 min using a Quorum Q150R ES coating device (Quorum Technologies, UK). Imaging was performed at various magnifications to analyze the microstructural properties of the films (Sabzi and Anijdan 2019).

### 2.15 Preparation of Chicken Meat Samples

Chicken fillet was purchased and transported to the laboratory under chilled conditions using ice packs. The visible fat was trimmed off, and the chicken breast was cut into 2 × 2 cm pieces, which were then ground using a conventional meat grinder. Twenty grams of ground chicken breast were prepared, and the produced films were placed on the minced meat samples. These samples were stored at 4°C for a duration of 15 days (days 0, 5, 10, and 15), after which the necessary analyses were conducted (Hassanin et al. 2017); the total fat content was analyzed using the Soxhlet extraction method, while protein content was determined following AOAC standard protocols (AOAC 1995); approximately 5 g of minced chicken meat was homogenized with 3 mL of 0.04 M phosphate buffer (pH 6.8) using an IKA T25 Ultra‐Turrax homogenizer (Germany) at 13,500 rpm for 10 s. The homogenate was refrigerated at 4°C for 1 h and then centrifuged at 2240 g for 5 min using a Sigma 3‐16 L centrifuge (Germany). The supernatant was passed through Whatman No. 42 filter paper, and the absorbance was measured at 503 nm using a Shimadzu UV‐1800 spectrophotometer (Japan); around 10 g of ground chicken meat was mixed with 90 mL of boiled and cooled distilled water, homogenized at 13,500 rpm for 30 s using an IKA T25 Ultra‐Turrax homogenizer (Germany), and the pH was measured using a calibrated Hanna HI 2211 pH meter (Romania) (Sallam et al. 2004); 10 g of minced chicken were mixed with 2 g of magnesium oxide, 300 mL of distilled water, and glass beads in a Kjeldahl digestion flask, digested with a Behr Kjeldahl digestion unit (Germany), distilled into a boric acid solution (2%) containing a methyl orange indicator, titrated with 0.01 N sulfuric acid, and the total volatile nitrogen (TVN) was calculated using a standard equation. The peroxide value (PV) was determined by weighing 0.2 g of chicken meat with an HR‐200 balance (A&D Company, Japan), adding 8 mL of a chloroform‐methanol mixture (7:3), vortexing the mixture, adding 50 μL of ammonium thiocyanate, vortexing again, adding 50 μL of ferrous chloride, mixing further, and measuring absorbance at 500 nm using an LKB Biochrom 4050 spectrophotometer (Sweden) (IDF 1991); the oxidation stability of chicken fat was assessed by analyzing 2 g of fat using a Rancimat 893 Professional Metrohm system (Switzerland) at 121°C with an airflow of 20 L/h, and the induction time was recorded (Salajegheh et al. 2018; Yaghoubi et al. 2021).

### 2.16 Microbiological Tests

Ten grams of the sample were weighed, and serial dilutions were prepared starting with a 1:10 dilution, followed by additional dilutions (e.g., 1:100) prepared using the same method; 1 mL of the first dilution was transferred to two sterile Petri dishes, and 1 mL of the second dilution was added to two other sterile dishes; 15 mL of molten Plate Count Agar (Merck, Germany) were poured onto each plate, mixed, and allowed to solidify; a sterile control plate was also prepared; the plates were incubated upside down at 30°C for 72 h in an incubator (Shimaz, Iran); plates containing between 10 and 300 colonies were selected for counting. Baird‐Parker Agar (Merck, Germany) was used for culturing coagulase‐positive Staphylococcus, and after inoculation, plates were kept at room temperature for 30 min and then incubated at 37°C for 24 h; colonies were marked and re‐incubated for another 24 h at the same temperature; plates with up to 150 colonies were counted; distinct colonies appeared shiny black or gray, convex, and partially surrounded by a dark halo, with colony diameters ranging from 1 to 1.5 mm after 24 h and 1.5 to 2.5 mm after 48 h of incubation. For Salmonella detection, a portion of the sample was added to Buffered Peptone Water (Merck, Germany) and incubated at 37°C for 18 h; for enrichment, inoculated samples were transferred to Rappaport‐Vassiliadis broth with soya and Muller‐Kauffman tetrathionate broth with novobiocin; the Rappaport‐Vassiliadis broth was incubated at 41.5°C and Muller‐Kauffman broth at 37°C, both for 24 h; enriched cultures were streaked onto Xylose Lysine Deoxycholate Agar (XLD) and Brilliant Green Agar (BGA) plates, which were incubated at 37°C for 24 h; suspected colonies were sub‐cultured for confirmation, followed by biochemical and serological tests using anti‐sera. Lauryl Sulfate Tryptose broth was inoculated with the sample and incubated at 37°C for 24 to 48 h; gas production was monitored using Durham tubes; positive samples were transferred to EC broth and incubated in a water bath at 44°C for 24 to 48 h; for further confirmation, samples were inoculated into peptone water without indole, incubated at 44°C for 24 to 48 h, and treated with Kovac's reagent; the formation of a red color on the surface was indicative of *E. coli* (Hassanien et al. 2016; Shaltout et al. 2015; Yeleusizova et al. 2023).

### 2.17 Fourier‐Transform Infrared Spectroscopy (FTIR)

Chicken meat samples were placed inside the Fourier‐Transform Infrared Spectrometer for analysis, and the spectra were recorded within the wavenumber range of 4000 to 400 cm^−1^ with a resolution of 4 cm^−1^ (Deniz et al. 2018).

### 2.18 Statistical Analysis

The obtained results were analyzed using SPSS software. The *t*‐Test was employed to evaluate the microbiological test results of the encapsulated sample, while Duncan's test was used for other analyses. All tests were conducted with a significance level of *α* = 0.05. All experiments were carried out in triplicate (*n* = 3), and independent samples were used for each treatment and characterization test. Results are expressed as mean ± standard deviation.

## 3 Result and Discussion

### 3.1 SEM

**FIGURE 1:**
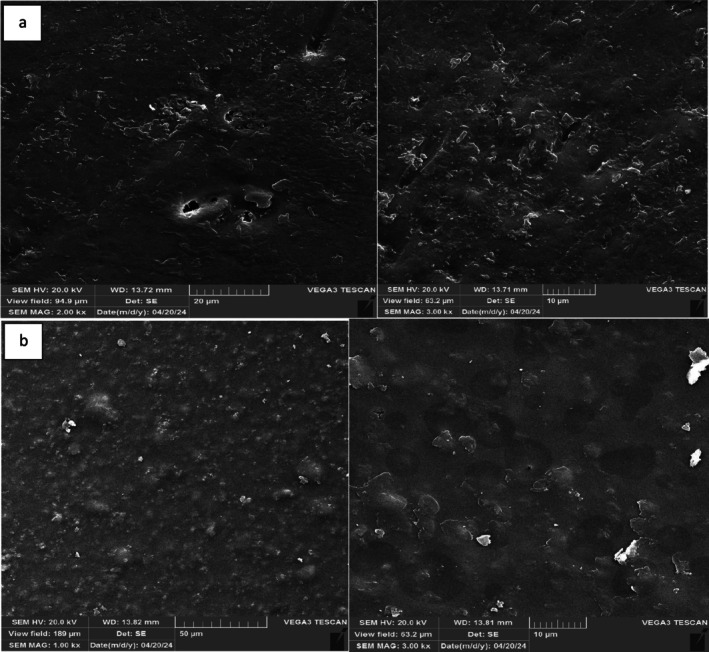
(a) Microscopic images of the free combination: Protein isolate and fiber extracted from wheat leaves with silver oxide, (b) Microscopic images of the encapsulated combination: Protein isolate and fiber extracted from wheat leaves with silver oxide.

The SEM (Figure 1) images depict the surface morphology of both free and encapsulated combinations of protein isolate, fiber, and silver oxide. In the free combination, the components appear as scattered, irregular particles with notable voids. This irregular structure likely arises from limited interactions between protein, fiber, and silver oxide due to the absence of an encapsulating framework, leading to heterogeneous particle dispersion. Such a structure may reduce stability and efficiency in applications like controlled release systems (Stanisz et al. 2020). In contrast, the encapsulated combination exhibits a more uniform and compact structure. The aggregation of particles and their encapsulation by the calcium chloride and sodium alginate matrix are distinctly visible, resulting in improved cohesion and reduced void spaces (Tsirigotis‐Maniecka et al. 2016). This indicates enhanced stability of the active components (e.g., silver oxide) within the encapsulated matrix. From a bonding perspective, in the encapsulated combination, ionic interactions between the carboxylate groups of alginate and calcium chloride, coupled with the physical entrapment of silver oxide particles, play a significant role (Milivojević et al. 2023). This structure is expected to enhance antibacterial efficacy and minimize premature degradation of silver oxide. Referring to the preparation process in the provided Table 1, the encapsulation procedure comprising injection into calcium chloride solution and hardening at low temperatures not only improves physical stability but also enhances functional properties through synergistic interactions between the components (Coetzee et al. 2020; Zhou et al. 2021). Thus, the SEM images clearly highlight the impact of encapsulation on improving the structure and uniformity of the combination. Previous studies have demonstrated that encapsulation using sodium alginate and calcium chloride enhances the stability of active materials and improves antibacterial properties, aligning with the findings of this study (Li et al. 2022). However, some research has reported that nanoparticle dispersion in free samples is more heterogeneous due to the lack of protective coating, which is consistent with the observations in our free combination samples (Nejad et al. 2015). Thus, the results of this study are in line with similar research in the field of encapsulation and the enhancement of bioactive compound properties.

### 3.2 Antioxidant Activity Measurements

The DPPH assay results (Table 3) demonstrate that the antioxidant activity of the encapsulated combination (protein isolate and fiber combined with silver oxide) is significantly higher than that of the free combination and control combination, as reflected by a lower IC50 value and a higher RSA percentage. The encapsulated combination, with an IC50 of 29.1 ± 0.9 μg/mL and RSA of 47.8% ± 1.3%, exhibits optimal antioxidant activity, which can be attributed to better stabilization of silver oxide within the alginate‐calcium matrix and stronger interactions between the encapsulated components (Lencina et al. 2012; Manzoor et al. 2022). In contrast, the free combination, with an IC50 of 43.5 ± 1.2 μg/mL and RSA of 31.2% ± 0.8%, shows lower antioxidant activity, likely due to the irregular dispersion of silver oxide and limited interactions among free components, a trend similarly reported in non‐encapsulated nanocomposite films by Roy and Rhim (2022). Additionally, the control sample, with an IC50 of 52.7 ± 1.5 μg/mL and RSA of 24.5% ± 0.6%, highlights that silver oxide alone, without stabilization in a biopolymer matrix, exhibits reduced antioxidant efficacy. From a bonding perspective, ionic interactions between the carboxylate groups of alginate and calcium ions in the encapsulated combination create a stable three‐dimensional network, effectively preventing premature degradation of silver oxide and enhancing antioxidant efficiency (Hurtado et al. 2022). Conversely, the absence of a protective coating in the free combination results in faster release and reduced antioxidative performance. These findings emphasize the advantages of encapsulation techniques in improving the stability and functional performance of bioactive and mineral components (Jafari et al. 2023). Protein isolate and fiber extracted from wheat leaves play pivotal roles as bioactive components in the formulations. Protein isolate, with its amino acid structure and functional groups such as amines and carboxylates, has a strong capacity to form hydrogen bonds and ionic interactions with other components, including alginate and silver oxide (Fernando et al. 2019; Rosiak et al. 2021). This contributes to the enhanced integrity of the encapsulation matrix. On the other hand, wheat fiber, due to its polymeric structure and high hydroxyl group content, acts as a biobased filler, forming three‐dimensional networks within the encapsulated matrix to improve mechanical and thermal stability (Pirzada et al. 2020). Furthermore, the hydrophilic nature of wheat fiber aids in moisture regulation and facilitates controlled release of active agents like silver oxide. The interactions between protein isolate and fiber, especially in the presence of calcium ions and alginate, lead to the formation of more stable structures. These structures not only prevent the premature degradation of silver oxide but also enhance antioxidant activity by promoting electron transfer and neutralizing free radicals (Wang et al. 2017). Thus, the presence of these two bioactive components in both free and encapsulated combinations is crucial for improving the antioxidant properties and structural stability of the formulations. Previous studies have demonstrated that the incorporation of protein isolate and fiber as bioactive components in encapsulated formulations significantly enhances stability and antioxidant activity (Wen et al. 2017; Zabot et al. 2022). These findings align with the results of this study, where the presence of fiber and protein improves matrix cohesion and functional properties (Javanmardi et al. 2021). However, some research has reported reduced dispersion and efficacy of active agents in the absence of encapsulation, consistent with the performance of our free combination samples.

**TABLE 3:** Antioxidant activity of free and encapsulated combinations (DPPH assay).

Sample type	Concentration (μg/mL)	IC50 (μg/mL)	RSA (%)
Free combination	50	43.5 ± 1.2^B^	31.2 ± 0.8^B^
Encapsulated combination	50	29.1 ± 0.9^A^	47.8 ± 1.3^A^
Control (silver oxide)	50	52.7 ± 1.5^C^	24.5 ± 0.6^C^

*Note:* The results are presented as mean ± standard deviation, and all experiments were conducted in triplicate. Differences in Latin letters indicate statistically significant differences at the 5% level.

### 3.3 Antimicrobial Activity

The antibacterial activity of free and encapsulated combinations against *Staphylococcus aureus* and *Escherichia coli* highlights the significant impact of encapsulation on enhancing the efficacy of the formulations (Table 4). The encapsulated combination demonstrated significantly higher inhibition zones (17.3 ± 0.8 mm for *Staphylococcus aureus* and 15.2 ± 0.5 mm for *Escherichia coli* ) compared to the free combination and the control combinations (*p* < 0.05). This enhanced antibacterial effect is likely due to the formation of a stable matrix by alginate and calcium ions, which stabilizes silver oxide and facilitates its controlled release (Islan et al. 2015). The presence of protein isolate and wheat fiber contributes further by forming hydrogen bonds and electrostatic interactions with bacterial cell walls, increasing membrane permeability and causing structural disruption of the cells (Liu et al. 2020; Meldrum et al. 2017). Ionic interactions between alginate carboxylate groups and calcium ions create a three‐dimensional network that enhances silver oxide's stability and prevents its premature degradation. In contrast, the free combination exhibited lower inhibition zones (10.5 ± 0.6 mm for *Staphylococcus aureus* and 8.7 ± 0.4 mm for *Escherichia coli* ), likely due to the irregular dispersion and faster degradation of silver oxide in the medium. The control (pure silver oxide) showed the lowest inhibition zones (9.1 ± 0.5 mm for *Staphylococcus aureus* and 7.8 ± 0.3 mm for *Escherichia coli* ), underlining the importance of stabilizing silver oxide within a biopolymeric matrix to enhance its efficacy. The differences in bacterial response between Gram‐positive and Gram‐negative strains are attributed to their cell wall structures, with the thicker peptidoglycan layer in Gram‐positive bacteria posing greater resistance to non‐encapsulated formulations (Chateau et al. 2020; Kalfopoulou 2019). The inclusion of protein isolate and fiber as bioactive components not only enhances physical and chemical interactions with silver oxide but also improves the overall antibacterial performance against both types of bacteria. Previous studies have shown that encapsulating silver oxide nanocomposites in biopolymeric matrices such as alginate enhances antibacterial activity due to controlled release and improved stability (Islan et al. 2015; Kraśniewska et al. 2020). The findings of this research align with these studies, as the encapsulated combination exhibited greater antibacterial efficacy compared to the free and control combinations. However, some studies have reported that in the absence of encapsulation, antibacterial efficiency declines due to irregular nanoparticle dispersion, which is consistent with the results observed in our free combination (Abadehie et al. 2021; Ebrahimi et al. 2020).

**TABLE 4:** Antibacterial activity of free and encapsulated combinations against *Staphylococcus aureus* and *Escherichia coli* .

Sample type	Bacteria	Inhibition zone diameter (mm)
Free combination	*Staphylococcus aureus*	10.5 ± 0.6^B^
	*Escherichia coli*	8.7 ± 0.4^B^
Encapsulated combination	*Staphylococcus aureus*	17.3 ± 0.8^A^
	*Escherichia coli*	15.2 ± 0.5^A^
Control (silver oxide)	*Staphylococcus aureus*	9.1 ± 0.5^C^
	*Escherichia coli*	7.8 ± 0.3^C^

*Note:* The results are presented as mean ± standard deviation, and all experiments were conducted in triplicate. Differences in Latin letters indicate statistically significant differences at the 5% level.

### 3.4 Analysis of the Produced Film

In Control Film, containing 5 g of protein and wheat leaf‐extracted fiber, the film structure demonstrates relative stability and adequate mechanical properties due to the presence of fiber and protein as active bio‐based components. However, the absence of silver oxide nanocomposites results in reduced antioxidant activity and diminished performance in tests such as FRAP (Bhutto et al. 2018; Flieger et al. 2021). This behavior is consistent with the general understanding that natural biopolymers without functional additives have limited oxidative stability and are more susceptible to environmental degradation. Zinina et al. (2023) similarly reported that alginate–protein films lacking metal‐based nanofillers show lower FRAP activity due to the absence of catalytic redox centers.

In non‐encapsulated Film, the addition of silver oxide nanocomposites to the protein–fiber film significantly enhances antioxidant activity and tensile strength, as the nanocomposites act as both antioxidant and antibacterial agents through gradual release within the film matrix (Vieira et al. 2022). However, the uneven distribution of nanoparticles leads to higher opacity and increased water absorption, indicating structural instability. Metha et al. (2024) confirmed that uncontrolled nanoparticle dispersion in alginate composites reduces hydrophobicity and increases water permeability, which agrees with the higher water‐absorption values observed in our non‐encapsulated samples.

In Encapsulated Film, the encapsulated combination of protein, fiber, and silver oxide nanocomposites utilizes alginate and calcium ions as encapsulating agents, forming a stable matrix that reduces opacity, increases contact angle, and decreases water absorption (Cofelice et al. 2019). This is supported by Zinina et al. (2023), who found that encapsulating metal nanocomposites within alginate–protein matrices leads to a compact, cross‐linked structure through ionic interactions between carboxylate and Ca^2+^ groups. Such networks provide uniform particle dispersion and controlled nanoparticle release, resulting in better optical transparency and mechanical strength. Furthermore, the encapsulated film exhibits superior mechanical and thermal performance due to ionic interactions between alginate, protein, and silver oxide nanocomposites, as evidenced by improved thickness and tensile strength. The significant differences between samples across various tests, particularly in antioxidant activity and contact angle, confirm the positive impact of encapsulation on film performance (Noronha et al. 2014; Vidal et al. 2024). Zinina et al. (2023) and Kousheh et al. (2020) observed similar improvements in fiber‐reinforced biofilms, attributing them to the high hydroxyl content of plant fibers, which enhances hydrogen bonding and mechanical cohesion within the matrix.

Overall, Encapsulated Film shows the best results across all evaluations (Table 5) due to its structural stability, controlled nanoparticle release, and stronger interactions among the components. Previous studies have shown that encapsulating metallic nanocomposites like silver oxide in biopolymeric matrices such as alginate enhances stability and controlled release, leading to improved antioxidant and mechanical properties of films (Alavi and Rai 2019; Pandey and Ramontja 2016). These findings align with our results, as the encapsulated film demonstrated superior thickness, contact angle, and reduced water absorption. However, some research has reported that uneven dispersion of nanoparticles in non‐encapsulated films decreases performance and increases opacity, which is consistent with our non‐encapsulated film results (Danilaev et al. 2023). Additionally, similar studies have confirmed that the inclusion of bio‐extracted fibers enhances structural stability and antioxidant effects (Kousheh et al. 2020).

**TABLE 5:** Evaluation of physical and chemical properties of control, non‐encapsulated, and encapsulated film samples.

Property	Control film	Non‐encapsulated film	Encapsulated film
FRAP (μmol Fe^2+^/g)	43.2 ± 1.8^C^	65.4 ± 2.1^B^	92.7 ± 3.4^A^
Thickness (mm)	0.14 ± 0.01^C^	0.16 ± 0.01^B^	0.21 ± 0.02^A^
Opacity (A_600_/cm)	5.3 ± 0.3^C^	4.3 ± 0.2^B^	3.1 ± 0.1^A^
Water solubility (%)	64.5 ± 2.7^C^	52.8 ± 2.5^B^	34.3 ± 1.8^A^
Moisture content (%)	22.4 ± 0.9^C^	18.7 ± 0.8^B^	15.2 ± 0.5^A^
Contact angle (°)	37.8 ± 1.2^C^	47.6 ± 1.3^B^	62.1 ± 1.9^A^
Tensile strength (MPa)	8.7 ± 0.5^C^	12.5 ± 0.7^B^	18.9 ± 0.9^A^
Water absorption (%)	72.4 ± 2.8^C^	67.2 ± 2.3^B^	49.6 ± 1.7^A^

*Note:* The results are presented as mean ± standard deviation, and all experiments were conducted in triplicate. Differences in Latin letters indicate statistically significant differences at the 5% level. Control Film: Film containing 5 g of protein, extracted fiber from wheat leaves per 100 mL of solution. Non‐Encapsulated Film: Film containing 5 g of protein‐extracted fiber and silver oxide nanocomposites per 100 mL of solution. Encapsulated Film: Encapsulated film containing 5 g of protein‐extracted fiber and silver oxide nanocomposites per 100 mL of solution.

### 3.5 Colorimetric Analysis of the Produced Film

The differences in the chemical and physical properties of control, non‐encapsulated, and encapsulated films can be attributed to the presence of silver oxide nanocomposites and the encapsulation process. In control film, containing only protein and fiber extracted from wheat leaves, the chemical interactions are limited to hydrogen bonds and van der Waals forces between protein and fiber molecules (Wongniramaikul et al. 2018). This results in a surface with lower uniformity and weaker color distribution, as reflected in the colorimetric indices (L*, a*, b*) (Table 6). In the non‐encapsulated film, the addition of silver oxide nanocomposites introduces electrostatic interactions and weak covalent bonding between nanocomposites and biomolecules. This modification increases surface density, decreases greenish shades (a*), and enhances brightness (L*) and yellowness (b*), but the aggregation of nanoparticles in certain areas reduces overall film uniformity (Fernandes et al. 2020). This pattern is consistent with reports by Moteriya and Chanda (2017) and Bothra et al. (2013) showing that insufficiently stabilized Ag/Ag_2_O in biopolymer matrices leads to local agglomeration, increased scattering, and hence spatially non‐uniform colorimetric response in starch/alginate systems.

**TABLE 6:** Colorimetric analysis (L*, a*, b*) of film samples.

Factor	Control film	Non‐encapsulated film	Encapsulated film
L*	50.0 ± 1.5^Aa^	70.0 ± 1.0^Bb^	90.0 ± 0.5^C^
a*	−3.0 ± 0.2^Ac^	−1.5 ± 0.1^Bb^	−0.5 ± 0.1^Ca^
b*	5.0 ± 0.3^Ac^	7.0 ± 0.2^Bb^	10.0 ± 0.1^Ca^

*Note:* The results are presented as mean ± standard deviation, and all experiments were conducted in triplicate. Differences in Latin letters indicate statistically significant differences at the 5% level Different letters (A, B, C) indicate statistically significant differences (*p* < 0.05) within each column. Control Film: Film containing 5 g of protein, extracted fiber from wheat leaves per 100 mL of solution. Non‐Encapsulated Film: Film containing 5 g of protein‐extracted fiber and silver oxide nanocomposites per 100 mL of solution. Encapsulated Film: Encapsulated film containing 5 g of protein‐extracted fiber and silver oxide nanocomposites per 100 mL of solution.

In the encapsulated film, the encapsulation process forms a more stable three‐dimensional network of alginate, distributing nanoparticles uniformly within the matrix. This uniformity significantly increases brightness (L*), reduces greenish shades (a*), and enhances yellowness (b*) in a statistically significant manner. The observed differences in colorimetric indices are attributed to stronger ionic interactions, reduced nanoparticle aggregation, and increased intermolecular bonding from the encapsulation process. These structural and chemical changes, which improve mechanical, thermal, and optical properties, underscore the importance of encapsulation technology and the effects of nanoparticles in enhancing film performance (Jafarzadeh and Jafari 2021; Singh et al. 2024). Studies by Kiseleva et al. (2020) and Lin et al. (2015) have demonstrated that incorporating metallic nanoparticles into bio‐matrices improves surface uniformity and optical properties, aligning with the results of our non‐encapsulated and encapsulated films. Elgamouz et al. (2021) emphasized the importance of encapsulation in preventing nanoparticle aggregation and enhancing colorimetric indices, which is also validated in our encapsulated film. However, some studies, such as Duarte et al. (2021), reported that nanoparticle aggregation in non‐encapsulated films could impair optical properties, as observed in our non‐encapsulated film.

**FIGURE 2:**
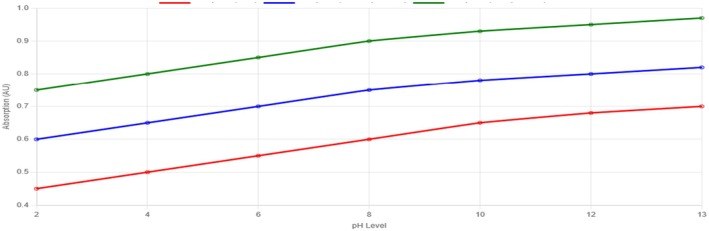
Colorimetric absorption analysis of indicator films at different pH levels. Red color: Control film, containing 5 g of protein, extracted fiber from wheat leaves per 100 mL of solution. Blue color: Non‐encapsulated film, containing 5 g of protein‐extracted fiber and silver oxide nanoparticles per 100 mL of solution. Green color: Encapsulated film containing 5 g of protein‐extracted fiber and silver oxide nanoparticles per 100 mL of solution.

In the conducted experiments, the observed color changes in the various films (control, non‐encapsulated, and encapsulated) were significantly influenced by the molecular structure of the films and the presence of silver oxide nanocomposites (Figure 2). In acidic conditions (low pH), the film samples exhibited orange and darker brown colors due to the protonation of functional groups such as carboxyl (—COOH) and amine (—NH2) present in the proteins and wheat fibers (Rashad et al. 2022; Yadav et al. 2024). This protonation altered the distribution of electrons and the absorption of light at different wavelengths. In non‐encapsulated and encapsulated films, the presence of silver oxide nanocomposites led to redox reactions on the nanoparticle surface, forming specific colored compounds (e.g., Ag^+^ and Ag^0^), which intensified the color. Comparable protonation‐induced chromatic shifts were described by Juneja et al. (2016), who linked the orange coloration of Ag_2_O nanocomposites at low pH to the formation of Ag^+^ species. In alkaline conditions (high pH), the increased OH^−^ ions caused deprotonation of the functional groups, creating negative charges on the film surface (Dong et al. 2021; Yu et al. 2014). This process was associated with the formation of ionic complexes between silver oxide nanocomposites and carboxylate groups. These complexes shifted the color of the films toward gray and green tones in non‐encapsulated and encapsulated films. In control film, the color change was limited due to the weak interactions between the fibers and proteins, and the absence of nanoparticles resulted in reduced uniformity in color absorption (Liu et al. 2022; Samyn et al. 2018; Zahiri et al. 2020). In non‐encapsulated film, the silver oxide nanocomposites directly interacted with the protein and fiber matrix, but aggregation of the nanoparticles in certain regions led to non‐uniform color changes. In encapsulated film, the encapsulation process created a stable three‐dimensional network, resulting in a more uniform distribution of nanoparticles and improved color intensity and uniformity across all pH levels. Table 7 demonstrates that the encapsulated film exhibited the highest light absorption (L*) across all pH levels, indicating better stability and uniformity of color in this film sample. Non‐encapsulated film displayed moderate absorption, likely due to nanoparticle aggregation and incomplete encapsulation. Control film, with the lowest absorption, reflected weaker chemical interactions and lower color stability. The statistical differences observed (*p* < 0.05) confirm that the presence of nanoparticles and the encapsulation process had a significant impact on the optical and chemical behavior of the films. Key chemical reactions included protonation, deprotonation, ionic complex formation, and changes in the oxidation state of silver oxide nanocomposites, all of which contributed to the observed color variations (Fernando and Zhou 2019; Molleman and Hiemstra 2015). These findings highlight the critical role of encapsulation and the incorporation of nanoparticles in modulating the optical and chemical properties of bio‐based films, which can be highly beneficial in industrial and biological applications. The study by Juneja et al. (2016) revealed that silver oxide exhibits orange‐red coloration in acidic pH and gray‐green in alkaline pH, which aligns with the color changes observed in our film samples. Furthermore, research by (Duraisamy et al. 2025); He et al. (2012) confirmed that color variations across pH levels are driven by changes in the oxidation state from Ag^+^ to Ag^0^ and the formation of ionic complexes, a mechanism also observed in our study. However, findings by (Fernando and Zhou 2019) indicated that nanoparticle aggregation at higher pH levels can sometimes reduce color intensity, consistent with our non‐encapsulated film results.

**TABLE 7:** Colorimetric absorption analysis of indicator films at different pH levels.

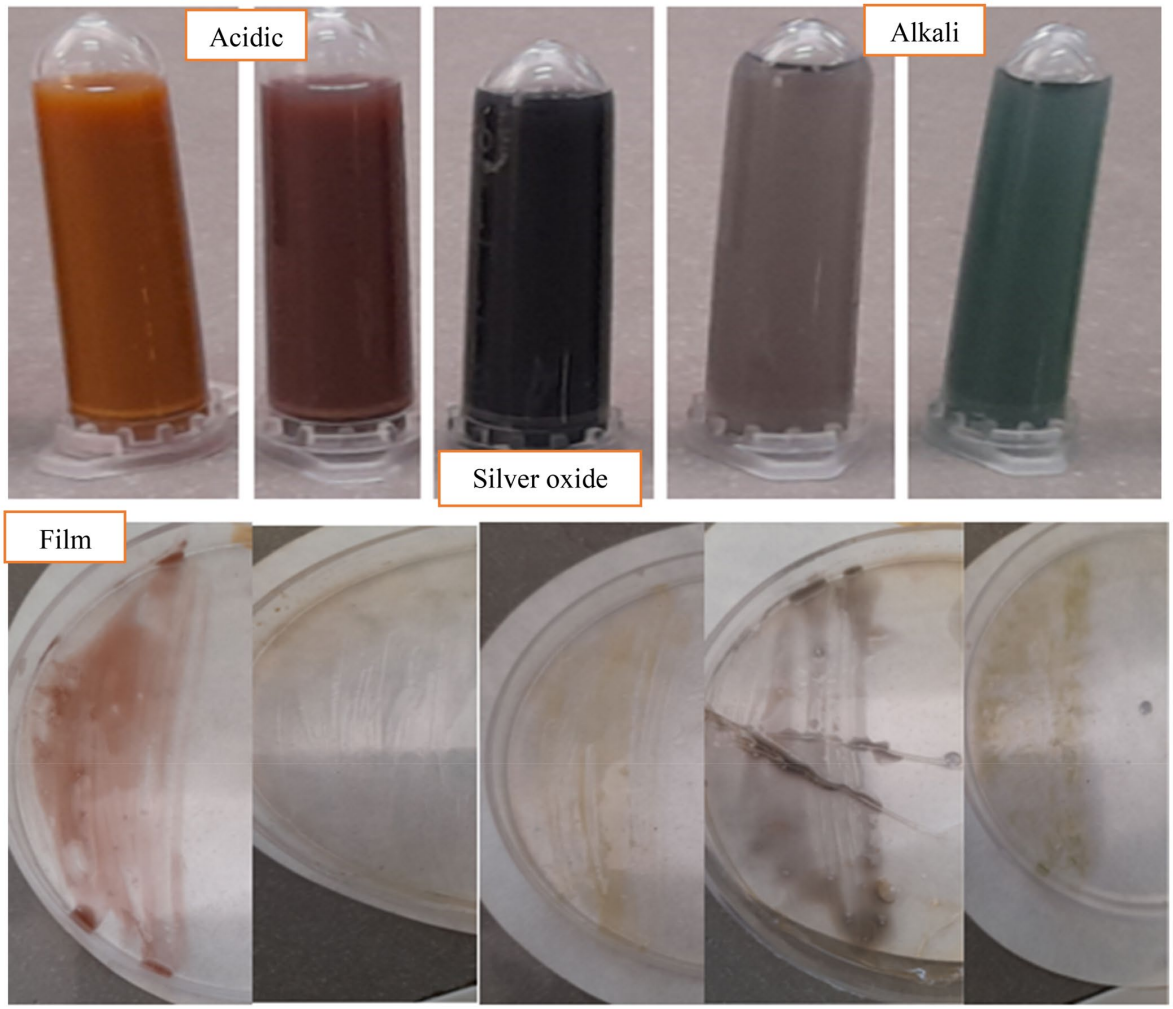			
pH	Control	Non‐encapsulated	Encapsulated
2	0.45 ± 0.02^A^	0.60 ± 0.03^B^	0.75 ± 0.01^C^
4	0.50 ± 0.03^A^	0.65 ± 0.02^B^	0.80 ± 0.02^C^
6	0.55 ± 0.02^A^	0.70 ± 0.01^B^	0.85 ± 0.01^C^
8	0.60 ± 0.02^A^	0.75 ± 0.02^B^	0.90 ± 0.02^C^
10	0.65 ± 0.03^A^	0.78 ± 0.02^B^	0.93 ± 0.01^C^
12	0.68 ± 0.02^A^	0.80 ± 0.03^B^	0.95 ± 0.02^C^
13	0.70 ± 0.03^A^	0.82 ± 0.02^B^	0.97 ± 0.01^C^

*Note:* The results are presented as mean ± standard deviation, and all experiments were conducted in triplicate. Differences in Latin letters indicate statistically significant differences at the 5% level. Different letters (A, B, C) indicate statistically significant differences (*p* < 0.05) within each column. Control Film: Film containing 5 g of protein, extracted fiber from wheat leaves per 100 mL of solution. Non‐Encapsulated Film: Film containing 5 g of protein‐extracted fiber and silver oxide nanocomposites per 100 mL of solution. Encapsulated Film: Encapsulated film containing 5 g of protein‐extracted fiber and silver oxide nanocomposites per 100 mL of solution.

### 3.6 FTIR

**FIGURE 3:**
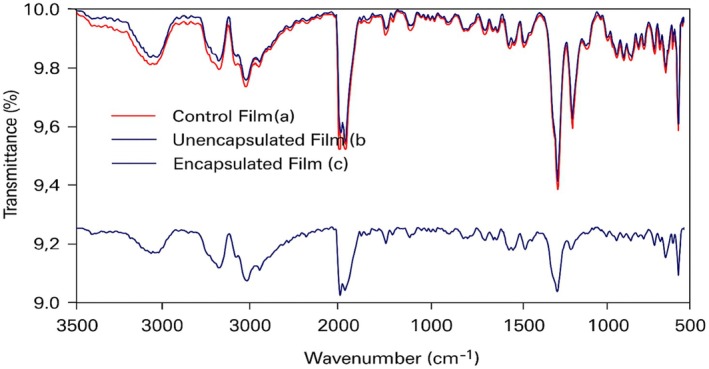
FTIR analysis of films (a) film containing 5 g of protein, extracted fiber from wheat leaves per 100 mL of solution, (b) film containing 5 g of protein‐extracted fiber and silver oxide nanoparticles per 100 mL of solution, (c) encapsulated film containing 5 g of protein‐extracted fiber and silver oxide nanoparticles per 100 mL of solution.

The FTIR spectrum of control film, comprising protein and wheat leaf‐extracted fiber (Figure 3), reveals the presence of key functional groups such as O—H stretching vibrations in the 3200–3400 cm^−1^ range, N—H bending in the 1500–1600 cm^−1^ region, and C—H stretching in the 2800–2950 cm^−1^ range, all characteristic of protein and fiber structures. The non‐encapsulated film, with added silver oxide nanocomposites, exhibits noticeable changes in the intensity of bands associated with O—H and N—H groups, indicating strong interactions between the nanoparticles and the functional groups of protein and fiber. The presence of nanoparticles is further confirmed by new bands in the 400–600 cm^−1^ region, corresponding to metal‐oxygen vibrations. Such Ag–O bands have been reported in alginate films containing Li–Ag_2_O nanocomposites, where incorporation led to additional bands around 500 cm^−1^ and changes in polymer–nanoparticle bonding (Giriyappa Thimmaiah et al. 2022).

In encapsulated film, the encapsulated film, the intensity and shifts in FTIR bands, particularly in the 3200–3400 and 1500–1600 cm^−1^ ranges, indicate stronger ionic and hydrogen bonding interactions among alginate, protein, and nanoparticles. These changes, alongside reduced O—H band intensity and increased C=O band intensities, suggest that encapsulation leads to a more stable and robust matrix (Ahmad and Ayub 2022; Riaz et al. 2018). The differences observed across the samples clearly demonstrate the role of nanoparticles and encapsulation in enhancing the chemical structure of the films, as stronger interactions among components reduce free groups and lead to the formation of more stable three‐dimensional networks. Furthermore, the encapsulated film exhibits distinct bands in the 1000–1500 cm^−1^ region, attributed to stretching vibrations of carboxylate groups and alginate‐related functionalities. These shifts confirm that encapsulation not only enhances chemical stability but also improves the functional properties of the films (Senathirajah et al. 2025; Steiner 2014).

### 3.7 TGA and DSC

**FIGURE 4:**
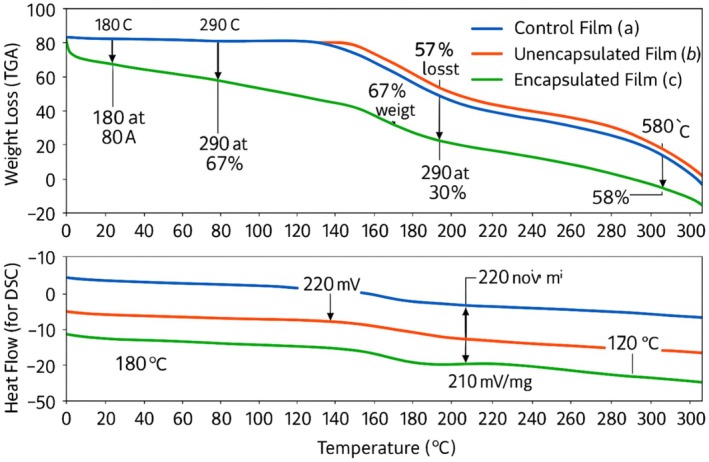
DSC and TGA analysis of (a) film containing 5 g of protein, extracted fiber from wheat leaves per 100 mL of solution, (b) film containing 5 g of protein‐extracted fiber and silver oxide nanoparticles per 100 mL of solution, (c) encapsulated film containing 5 g of protein‐extracted fiber and silver oxide nanoparticles per 100 mL of solution.

The TGA and DSC spectra of control, non‐encapsulated, and encapsulated films (Figure 4) reveal significant differences in thermal stability and heat transfer behavior. In the control film, containing protein and wheat leaf‐extracted fiber, an initial weight loss below 100°C is observed due to the loss of absorbed moisture. This weight loss is attributed to the bio‐matrix structure and the presence of polar groups retaining moisture. Between 200 to 300°C, further weight loss occurs, corresponding to the thermal decomposition of fiber and protein (Zhu 2015). This two‐stage pattern is widely reported for natural‐polymer films and cellulosic/alginate systems and matches our thermograms.

In non‐encapsulated film, which incorporates silver oxide nanocomposites, improved thermal stability is evident, as stronger interactions between the nanoparticles and the bio‐matrix are established. Weight loss in this sample is more gradual, and DSC peaks corresponding to thermal transitions indicate an increased decomposition temperature of organic components (Chaturvedi and Dave 2013). The nanoparticles, acting as reinforcing agents, reduce the degradation rate and enhance structural stability. Our DSC endo/exotherms likewise shift to higher temperatures, consistent with delayed thermal transitions reported for Ag‐containing polymer matrices. In encapsulated film, the encapsulated formulation, thermal behavior shows even greater improvement. The initial weight loss is lower, indicating better moisture retention within the more stable matrix. Furthermore, the main decomposition temperature is higher than in control and non‐encapsulated film, reflecting increased resistance to thermal degradation due to encapsulation. The exothermic and endothermic peaks in the DSC spectrum of encapsulated film indicate more complex thermal transitions and better energy stability, attributed to ionic interactions among alginate, nanoparticles, and proteins (Leyva‐Porras et al. 2019). These analyses clearly demonstrate that the presence of nanoparticles and the encapsulation process significantly enhance thermal stability and heat transfer properties. The presence of fiber and protein in control film, as a bio‐based matrix with polar structures, leads to moisture absorption and faster thermal degradation at lower temperatures, whereas in non‐encapsulated film, the addition of silver oxide nanocomposites creates stronger interactions between the bio‐matrix's polar groups and nanoparticles, improving thermal stability and heat transfer. Encapsulated film, with encapsulated components, not only reduces moisture absorption and increases decomposition temperature but also exhibits more stable and complex thermal transitions in the DSC spectrum due to the formation of a stronger ionic network within the matrix. These findings demonstrate that encapsulation and nanoparticle incorporation not only enhance chemical and mechanical properties but also significantly improve thermal stability and energy transfer kinetics (Nordi et al. 2025). The FTIR spectrum reveals stronger chemical interactions between functional groups of protein, fiber, and silver oxide nanocomposites in non‐encapsulated and encapsulated films, which are corroborated by TGA results showing improved thermal stability and reduced degradation rates. In encapsulated film, encapsulation and ionic bonding among alginate, nanoparticles, and protein reduce moisture absorption (FTIR), increase decomposition temperature (TGA), and exhibit more complex thermal transitions in the DSC spectrum (El‐Houssiny et al. 2016). Overall, encapsulation and nanoparticle incorporation enhance chemical stability (FTIR), thermal resistance (TGA), and heat transfer behavior (DSC) through the formation of more stable three‐dimensional networks and stronger intercomponent interactions. Previous studies, such as Ahina et al. (2023) and Sreekumar and Kiran (2024), have demonstrated that incorporating metallic nanoparticles into bio‐matrices enhances thermal stability and mechanical properties, which aligns well with our TGA and DSC results. Additionally, research by Morales et al. (2020) confirmed that encapsulation of bio‐based components reduces moisture absorption and improves thermal transitions, consistent with our FTIR and DSC findings. However, some studies, like Souza et al. (2023), reported that nanoparticle dispersion in non‐encapsulated films can decrease matrix integrity, which corresponds to the behavior observed in our non‐encapsulated film.

### 3.8 SEM

**FIGURE 5:**
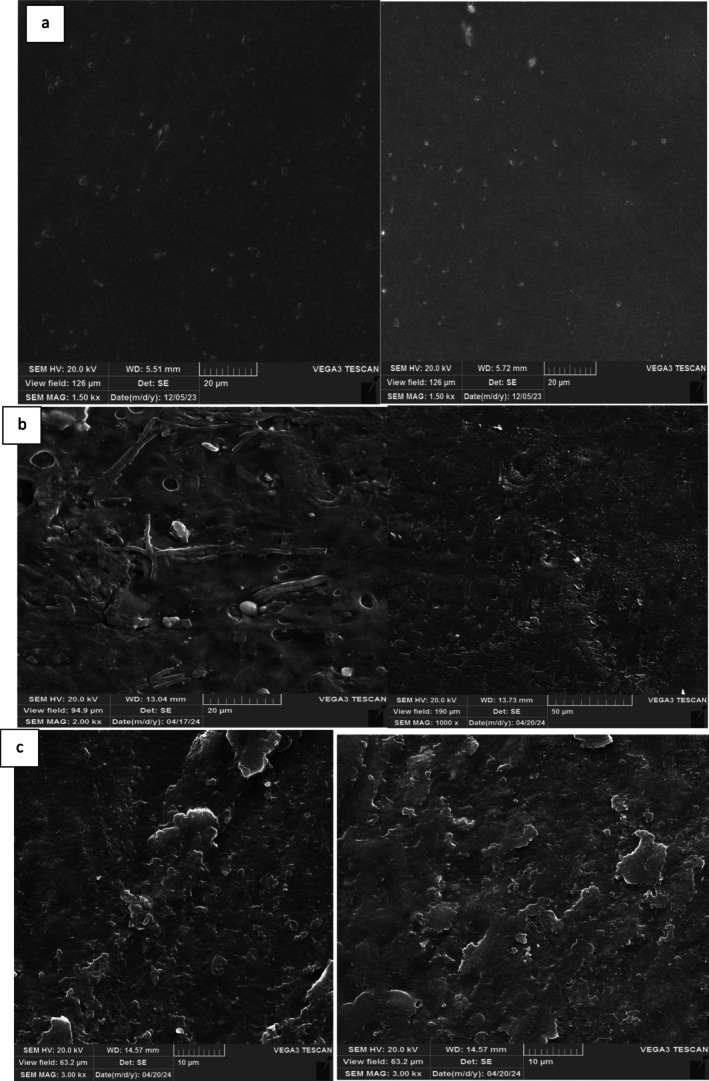
SEM images of (a) control film: Film containing 5 g of protein, extracted fiber from wheat leaves per 100 mL of solution; (b) non‐encapsulated film: Film containing 5 g of protein‐extracted fiber and silver oxide nanoparticles per 100 mL of solution; (c) encapsulated film containing 5 g of protein‐extracted fiber and silver oxide nanoparticles per 100 mL of solution.

SEM images of control, non‐encapsulated, and encapsulated films (Figure 5) reveal significant differences in surface morphology and component distribution. In control film, composed of protein and wheat leaf‐extracted fiber, the film surface exhibits a clearly homogeneous structure with relatively low density, attributed to the absence of nanoparticles and limited interactions among matrix components (Zena et al. 2023). This structure shows evenly distributed fibers, but the presence of small voids and fine pores is noticeable, resulting from the gaps between protein and fiber chains (Fernando et al. 2019; Khorshidi et al. 2016). In non‐encapsulated film, containing silver oxide nanocomposites, the SEM images display an increase in surface density and the presence of nanoparticles as brighter spots on the film surface. This increased density is linked to interactions between nanoparticles and fibers, as well as the enhanced structure of the matrix. However, the dispersion of nanoparticles appears irregular in some areas, forming aggregates that could reduce film transparency and increase opacity. Similar agglomeration behavior has been described in Ag_2_O‐filled starch and alginate nanocomposites, where partial aggregation reduces surface homogeneity and creates microscale roughness, affecting both optical and mechanical performance (Mahlambi and Moloto 2022).

In encapsulated film, the encapsulated film, SEM images demonstrate a highly uniform surface structure with evenly distributed nanoparticles within the matrix. This uniformity is attributed to the use of alginate in the encapsulation process, which forms a stable three‐dimensional network and promotes stronger ionic interactions among components. The surface of the encapsulated film lacks large pores and scattered spots, indicating greater stability and more uniform nanoparticle distribution (Ahmad and Ayub 2022; Barreneche et al. 2018). Moreover, encapsulation enhances interfacial adhesion between polymer chains and inorganic particles, minimizing phase separation and improving dispersion stability. These structural improvements explain the higher tensile strength and barrier performance observed in our encapsulated films, consistent with reports by Cano‐Vicent et al. (2023), where uniform nanoparticle embedding increased compactness and reduced porosity.

The observed differences among the samples highlight the positive impact of nanoparticles and the encapsulation process in improving surface morphology, uniformity, and matrix stability. These changes can explain the enhanced mechanical, thermal, and functional properties of the films. Previous studies have demonstrated that adding metallic nanoparticles like silver oxide to bio‐matrices improves mechanical, thermal, and surface uniformity properties (Akceoglu et al. 2021; Sarwar et al. 2018). These findings align with our observations of non‐encapsulated and encapsulated films, where nanoparticles and encapsulation enhanced distribution and surface uniformity. Additionally, research such as Ayyaril et al. (2023) reported that improper dispersion of nanoparticles in non‐encapsulated films can lead to aggregation and reduced stability, which was also evident in our non‐encapsulated film. Similar studies by Atinafu et al. (2020) confirmed that encapsulation forms more stable three‐dimensional networks and prevents large pore formation, consistent with our encapsulated film results.

### 3.9 Effect of Film on Chicken Meat

The effect of three types of coated films on the chemical and quality attributes of chicken meat during a 15‐day storage period was investigated. Control film, containing 5 g of protein and wheat leaf‐extracted fiber per 100 mL solution, served as the base film. Due to its hydrophilic structure, this film exhibited limited effects on reducing lipid oxidation and pH changes, likely attributed to its high oxygen and moisture permeability. Non‐encapsulated film, comprising 5 g of protein and fiber with silver oxide nanocomposites, demonstrated superior performance in reducing peroxide values (PV) and total volatile nitrogen (TVN), attributed to the antimicrobial properties of the nanoparticles. Silver oxide disrupted bacterial membranes and reduced microbial activity, thereby preventing the formation of nitrogenous compounds and secondary peroxides (Durán et al. 2016; Huang et al. 2020; Quinteros et al. 2016). Similar findings were reported by Carbone et al. (2016), who showed that silver nanocomposites embedded in polymeric films significantly reduced oxidative rancidity and microbial spoilage in fresh meat packaging. Likewise, Khan et al. (2022) demonstrated that pullulan films containing silver nanocomposites maintained lipid stability and prevented off‐flavor formation in broiler meat. These studies confirm that the antimicrobial and antioxidant mechanisms of silver nanocomposites are primarily due to their ability to generate reactive oxygen species that damage microbial membranes and to chelate free radicals, thus retarding lipid oxidation.

Furthermore, the reduction in turbidity and enhanced oxidation stability in non‐encapsulated film indicated the direct impact of nanoparticles in inhibiting lipid oxidation processes. Encapsulated film, an encapsulated film containing silver oxide nanocomposites exhibited the highest levels of stability and quality preservation. Encapsulation of nanoparticles within the polymeric matrix reduced their direct migration to the meat and provided controlled release, leading to a gradual increase in antimicrobial and antioxidant activity over the storage period. Encapsulation has been reported to enhance nanoparticle functionality by preventing particle agglomeration and allowing sustained antimicrobial activity. E Abo‐Gaball et al. (2022) found that nanoparticle‐reinforced edible coatings effectively delayed microbial growth and lipid oxidation in chicken fillets. The encapsulation matrix can thus act as both a physical barrier and a carrier system that releases nanoparticles in a controlled manner, which improves both the safety and shelf‐life of the product.

Results revealed that pH changes in nanoparticle‐containing films were less pronounced compared to the base film, attributed to reduced microbial activity and acid production. Additionally, a significant reduction in metmyoglobin levels in non‐encapsulated and encapsulated films reflected decreased pigment oxidation and sustained the natural color of the meat over time (Fu et al. 2017; Liu et al. 2015). Overall, the encapsulated film demonstrated the most effective preservation of chicken meat quality (Table 8), with significant reductions in TVN, PV, and metmyoglobin compared to other samples. This was attributed to the controlled release of nanoparticles and simultaneously retained antimicrobial and antioxidant properties. These findings underscore that the incorporation of silver oxide nanocomposites with an encapsulation approach not only enhances meat quality during storage but also holds great potential for the development of smart and active food packaging solutions.

**TABLE 8:** Effect of coated films on evaluation of chemical properties of chicken meat during storage.

Factors	Units	Storage time (Days)	Control film	Non‐encapsulated film	Encapsulated film
Fat Content	% w/w	0	5.2 ± 0.1^Aa^	5.2 ± 0.1^Aa^	5.2 ± 0.1^Aa^
		5	5.7 ± 0.2^Ab^	5.4 ± 0.1^Bb^	5.3 ± 0.1^Cb^
		10	6.1 ± 0.3^Ac^	5.8 ± 0.2^Bc^	5.5 ± 0.1^Cc^
		15	6.5 ± 0.4^Ad^	6.1 ± 0.2^Bd^	5.7 ± 0.2^Cd^
pH	—	0	5.8 ± 0.1^Aa^	5.8 ± 0.1^Aa^	5.8 ± 0.1^Aa^
		5	6.1 ± 0.2^Ab^	5.9 ± 0.1^Bb^	5.8 ± 0.1^Cb^
		10	6.4 ± 0.2^Ac^	6.2 ± 0.1^Bc^	6.0 ± 0.1^Cc^
		15	6.7 ± 0.3^Ad^	6.4 ± 0.2^Bd^	6.1 ± 0.1^Cd^
TVN	mg N/100 g	0	10.5 ± 0.5^Aa^	10.5 ± 0.5^Aa^	10.5 ± 0.5^Aa^
		5	15.2 ± 0.6^Ab^	12.8 ± 0.4^Bb^	11.5 ± 0.3^Cb^
		10	21.8 ± 0.8^Ac^	18.4 ± 0.6^Bc^	15.2 ± 0.5^Cc^
		15	29.4 ± 1.0^Ad^	24.5 ± 0.8^Bd^	18.5 ± 0.6^Cd^
Peroxide Value	meq O_2_/kg	0	1.2 ± 0.1^Aa^	1.2 ± 0.1^Aa^	1.2 ± 0.1^Aa^
		5	2.5 ± 0.2^Ab^	2.0 ± 0.1^Bb^	1.8 ± 0.1^Cb^
		10	3.8 ± 0.3^Ac^	2.8 ± 0.2^Bc^	2.2 ± 0.1^Cc^
		15	5.1 ± 0.4^Ad^	3.6 ± 0.3^Bd^	2.8 ± 0.2^Cd^
Oxidation Stability	h	0	12.5 ± 0.8^Aa^	12.5 ± 0.8^Aa^	12.5 ± 0.8^Aa^
		5	10.8 ± 0.7^Ab^	11.5 ± 0.6^Bb^	11.8 ± 0.5^Cb^
		10	9.2 ± 0.6^Ac^	10.2 ± 0.5^Bc^	11.2 ± 0.4^Cc^
		15	7.5 ± 0.5^Ad^	9.2 ± 0.4^Bd^	10.5 ± 0.3^Cd^
Metmyoglobin	%	0	15.5 ± 0.9^Aa^	15.5 ± 0.9^Aa^	15.5 ± 0.9^Aa^
		5	22.3 ± 1.1^Ab^	19.8 ± 1.0^Bb^	18.5 ± 0.8^Cb^
		10	30.2 ± 1.3^Ac^	25.5 ± 1.2^Bc^	22.8 ± 1.0^Cc^
		15	38.5 ± 1.5^Ad^	31.2 ± 1.3^Bd^	26.5 ± 1.2^Cd^

*Note:* The results are presented as mean ± standard deviation, and all experiments were conducted in triplicate. Differences in Latin letters indicate statistically significant differences at the 5% level Different letters (A, B, C) indicate statistically significant differences (*p* < 0.05) within each column. Control Film: Film containing 5 g of protein, extracted fiber from wheat leaves per 100 mL of solution. Non‐Encapsulated Film: Film containing 5 g of protein‐extracted fiber and silver oxide nanocomposites per 100 mL of solution. Encapsulated Film: Encapsulated film containing 5 g of protein‐extracted fiber and silver oxide nanocomposites per 100 mL of solution.

### 3.10 Microbiological Tests

This study evaluated the effect of three types of coated films on the microbial indices of chicken meat during a 15‐day storage period. Control film, the base film containing 5 g of protein and wheat leaf‐extracted fiber per 100 mL solution, exhibited higher microbial growth, particularly *Staphylococcus aureus* and *Escherichia coli* , compared to other samples due to its hydrophilic structure and high permeability to moisture and oxygen. Non‐encapsulated and encapsulated films, which incorporated silver oxide nanocomposites, demonstrated superior antimicrobial activity. In non‐encapsulated film, silver oxide nanocomposites exhibited their antimicrobial effects through direct release, disrupting bacterial cell membranes and reducing metabolic activity, resulting in a significant decrease in CFU/g for *Escherichia coli* and *Staphylococcus aureus* (Table 9) (Cao et al. 2019; Liu et al. 2025). However, the rapid migration of nanoparticles from the film to the meat surface limited their long‐term efficacy. In encapsulated film, nanoparticles were embedded within the polymeric matrix of plant fiber, allowing controlled release and gradual inhibition of microbial growth, which was significantly evident in the reduction of *Staphylococcus aureus* and *Escherichia coli* . Furthermore, results for Salmonella revealed its significant presence in control film from day 5 onward, while it was entirely absent in non‐encapsulated and encapsulated films throughout the storage period, confirming the efficacy of silver oxide nanocomposites in preventing Salmonella contamination. The significant reduction in microbial load in encapsulated film was attributed to the synergy between encapsulation and the antimicrobial properties of nanoparticles, achieved through controlled migration to the surface and prolonged antimicrobial effects. Efficacy of silver oxide nanocomposites in reducing the growth of *Escherichia coli* and *Staphylococcus aureus* . Additionally, the study by Shetta et al. (2019) demonstrated that encapsulation of nanoparticles within a polymeric matrix reduced nanoparticle migration and enhanced antimicrobial efficacy, consistent with our results. However, compared to Vandeputte et al. (2017), which reported limited reductions in microbial load, our work showed greater effectiveness in controlling *Salmonella*. These results are in agreement with several recent studies that investigated the antimicrobial potential of silver nanocomposite‐based films in meat packaging systems. Similarly, Khan et al. (2022) reported that pullulan‐based films containing silver nanocomposites maintained microbiological quality of broiler meat for up to 15 days of storage. Moreover, Li et al. (2022) found that nanocomposite films with controlled nanoparticle release maintained antibacterial efficacy over extended storage periods, supporting the advantage of encapsulation in prolonging antimicrobial action. E Abo‐Gaball et al. (2022) further confirmed that edible films reinforced with nanoparticles completely inhibited Salmonella and *Listeria monocytogenes* during cold storage of poultry products. Together, these findings validate the hypothesis that encapsulated silver oxide nanocomposites offer enhanced and sustained protection against spoilage and pathogenic bacteria compared to conventional non‐encapsulated systems.

**TABLE 9:** Films coated on chicken meat and evaluation of their microbial properties during storage.

Microbial factors	Units	(Days)	Control film	Non‐encapsulated film	Encapsulated film
*Staphylococcus aureus* (Coagulase‐positive)	CFU/g	0	2.5 × 10^3^ ± 0.2^Aa^	2.5 × 10^3^ ± 0.2^Aa^	2.5 × 10^3^ ± 0.2^Aa^
		5	4.2 × 10^3^ ± 0.3^Ab^	3.1 × 10^3^ ± 0.2^Bb^	2.8 × 10^3^ ± 0.2^Cb^
		10	6.5 × 10^3^ ± 0.5^Ac^	4.5 × 10^3^ ± 0.3^Bc^	3.5 × 10^3^ ± 0.3^Cc^
		15	8.8 × 10^3^ ± 0.7^Ad^	5.8 × 10^3^ ± 0.4^Bd^	4.0 × 10^3^ ± 0.4^Cd^
*Escherichia coli*	CFU/g	0	1.8 × 10^2^ ± 0.1^Aa^	1.8 × 10^2^ ± 0.1^Aa^	1.8 × 10^2^ ± 0.1^Aa^
		5	3.0 × 10^2^ ± 0.2^Ab^	2.2 × 10^2^ ± 0.1^Bb^	2.0 × 10^2^ ± 0.1^Cb^
		10	5.0 × 10^2^ ± 0.3^Ac^	3.5 × 10^2^ ± 0.2^Bc^	2.5 × 10^2^ ± 0.2^Cc^
		15	7.5 × 10^2^ ± 0.4^Ad^	4.8 × 10^2^ ± 0.3^Bd^	3.0 × 10^2^ ± 0.2^Cd^
Salmonella	Presence/Absence	0	Absent (−)^Aa^	Absent (−)^Aa^	Absent (−)^Aa^
		5	Present (+)^Ab^	Absent (−)^Bb^	Absent (−)^Cb^
		10	Present (+)^Ac^	Absent (−)^Bc^	Absent (−)Cc
		15	Present (+)^Ad^	Absent (−)^Bd^	Absent (−)^Cd^

*Note:* The results are presented as mean ± standard deviation, and all experiments were conducted in triplicate. Differences in Latin letters indicate statistically significant differences at the 5% level Different letters (A, B, C) indicate statistically significant differences (*p* < 0.05) within each column. Control Film: Film containing 5 g of protein, extracted fiber from wheat leaves per 100 mL of solution. Non‐Encapsulated Film: Film containing 5 g of protein‐extracted fiber and silver oxide nanocomposites per 100 mL of solution. Encapsulated Film: Encapsulated film containing 5 g of protein‐extracted fiber and silver oxide nanocomposites per 100 mL of solution.

### 3.11 Fourier‐Transform Infrared Spectroscopy (FTIR)

**FIGURE 6:**
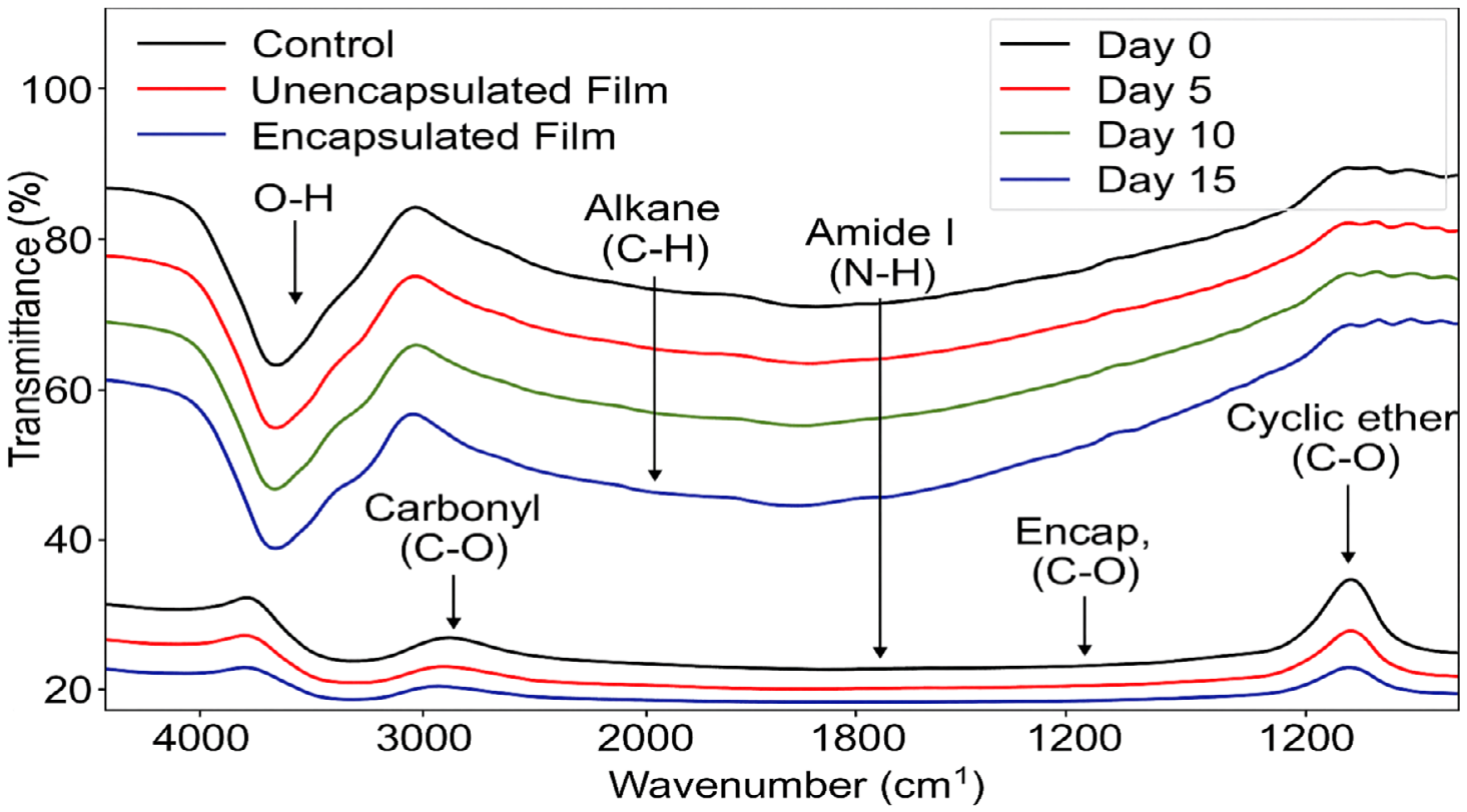
FTIR analysis of coated film on chicken meat during storage (a) film containing 5 g of protein, extracted fiber from wheat leaves per 100 mL of solution, (b) film containing 5 g of protein‐extracted fiber and silver oxide nanoparticles per 100 mL of solution, (c) encapsulated film containing 5 g of protein‐extracted fiber and silver oxide nanoparticles per 100 mL of solution.

The type of protein used in bio‐based films plays a critical role in determining their structure, stability, and functionality. Proteins, as natural biopolymers, contribute significantly to the mechanical and chemical properties of films due to their polar functional groups (e.g., amines and carbonyls) and their ability to form three‐dimensional networks through hydrogen bonding and cross‐linking (An et al. 2018). In this study, the use of proteins extracted from plant sources (e.g., wheat) enhanced the films' biodegradability and environmental compatibility. Proteins exhibit characteristic peaks in FTIR spectra due to amide (N—H) and carbonyl (C=O) groups, and the intensity and position of these peaks reflect structural changes and interactions with other components, such as fibers and nanoparticles. By forming matrix networks, proteins play a crucial role in controlling the permeability of films and reducing food oxidation. In non‐encapsulated and encapsulated films, strong interactions between silver oxide nanocomposites and the polar groups of proteins (e.g., hydroxyl and carbonyl) improved film stability and reduced microbial access to the film surface (Pasieczna‐Patkowska et al. 2025; Ridgley et al. 2013). In the encapsulated film, proteins provided a more stable matrix, reducing structural degradation and enabling controlled nanoparticle release. This feature contributed to preserving mechanical properties and extending the functional lifespan of the film. Therefore, the type of protein and its interactions with other components directly influence the final properties of the films and their protective performance on food products (Chen et al. 2019). The functional groups identified in the spectra (Table 10 and Figure 6) reveal critical insights. The hydroxyl group (O—H) peaks in the 3280–3300 cm^−1^ region indicate the presence of bound water and protein‐fiber interactions. In non‐encapsulated and encapsulated films, the decreased intensity of this peak in later days reflects stronger interactions between nanoparticles and the film matrix, reducing hydroxyl group accessibility. The carbonyl group (C=O) peaks in the 1730–1750 cm^−1^ region represent carbonyl stretching, which is more intense in control film but reduced in non‐encapsulated and encapsulated films due to the effects of nanoparticles. A slight shift toward lower wavenumbers indicates stronger hydrogen bonding between nanoparticles and polar groups in the film matrix (Ahmad et al. 2012; Kumari and Mohan 2025). In the 2920–2950 cm^−1^ region corresponding to alkane (C—H) stretching, the reduced intensity in nanoparticle‐containing samples signifies fewer free hydrocarbon chains and better interactions with nanoparticles. Amide (N—H) peaks in the 1640–1660 cm^−1^ region, representing protein bonds, show less reduction in encapsulated films, indicating greater structural stability. Additionally, cyclic ether (C—O) groups in the 1040–1100 cm^−1^ region show reduced intensity in non‐encapsulated and encapsulated films, attributed to interactions with nanoparticles (Hartrianti 2016; Hoppenreijs 2024). The impact of these films on chicken meat quality revealed that encapsulated films performed best due to controlled nanoparticle release, reduced microbial growth, and preservation of mechanical and chemical properties. In contrast, films without nanoparticles (control film) exhibited weaker performance due to reduced antibacterial properties and increased oxidation. Several similar studies have investigated antimicrobial bio‐based films and their impact on chicken meat quality. For example, a study by Shankar et al. (2021) demonstrated that films containing silver nanoparticles effectively inhibited microbial growth and reduced structural changes in functional groups (e.g., O—H and C=O) during storage. Additionally, research by Alaburdaitė and Krylova (2023) confirmed that the reduction in hydroxyl and carbonyl group intensities in FTIR spectra correlates with improved film stability and reduced oxidation. The findings of this study align with these works, highlighting the positive role of encapsulated films in preserving meat quality.

**TABLE 10:** FTIR spectral interpretation of functional groups in film samples.

Functional group	Wavenumber range (cm^−1^)	Sample observation	Interpretation
Hydroxyl (O—H)	3280–3300	Intense in Control, reduced in Unencapsulated and Encapsulated	Indicates bound water and protein‐fiber interactions. Reduction due to stronger matrix‐nanocomposites interactions
Carbonyl (C=O)	1730–1750	Shifted in nanocomposites‐containing films (Unencapsulated, Encapsulated)	Represents carbonyl stretching. Shift due to hydrogen bonding with nanoparticles
Alkane (C—H)	2920–2950	Reduced in Unencapsulated and Encapsulated	Suggests fewer free hydrocarbon chains due to interactions with nanoparticles
Amide I (N—H)	1640–1660	Stable in Encapsulated, slightly reduced in Unencapsulated	Indicates protein structure stability, better preserved in encapsulated samples
Cyclic Ether (C—O)	1040–1100	Reduced in nanoparticle‐containing samples	Represents interactions between ether groups and nanoparticles

These results are consistent with other recent investigations where silver nanocomposites were integrated into protein‐based and polysaccharide films. For instance, Kraśniewska et al. (2020) reported that silver nanocomposites incorporated into gelatin films led to reduced O—H and C=O stretching intensities, confirming the formation of hydrogen bonds between nanoparticles and biopolymer chains, which enhanced barrier properties and reduced oxygen permeability. Furthermore, Athanasopoulou et al. (2024) confirmed that nanoparticle addition caused lower C–H stretching intensity and higher network compactness, resulting in improved stability during storage of fish fillets.

Li et al. (2022) also highlighted that encapsulated nanocomposite films with metal nanoparticles exhibit less pronounced carbonyl peaks due to controlled nanoparticle release and reduced particle aggregation. Ahmed et al. (2018) demonstrated that silver‐based nanocomposite films used for chicken meat packaging significantly stabilized amide bands and lowered oxidative degradation during cold storage. These findings reinforce the current results by showing that the encapsulation of silver oxide nanocomposites leads to sustained molecular stability and antimicrobial performance throughout storage.

## 4 Conclusion

This study investigated the effects of bio‐based films containing silver oxide nanocomposites on the physical, chemical, thermal, and microbiological properties of various samples. The results demonstrated that the presence of nanoparticles and the encapsulation process significantly improved the mechanical properties, thermal stability, and antioxidant and antibacterial performance of the films. In the nanoparticle‐containing samples (non‐encapsulated and encapsulated films), stronger antibacterial effects were observed, attributed to the disruption of bacterial cell walls and the reduction of their metabolic activity. The encapsulated film (Sample 3) showed superior performance in preserving the quality of chicken meat, which was attributed to the controlled release of nanoparticles and reduced direct migration to the meat surface. FTIR analyses revealed strong interactions between nanoparticles, proteins, and fibers, leading to the formation of stable three‐dimensional networks that enhanced chemical properties and reduced moisture absorption. TGA and DSC results confirmed increased decomposition temperatures and improved thermal stability in nanoparticle‐containing samples. SEM images showed that encapsulation facilitated more uniform nanoparticle distribution within the film matrix, preventing aggregation, whereas in non‐encapsulated films, nanoparticle clustering reduced structural uniformity. Microbiological tests indicated that nanoparticle‐containing samples, especially the encapsulated film, significantly reduced the growth of pathogenic bacteria such as *Staphylococcus aureus* and *Escherichia coli* , and prevented Salmonella growth. During storage, the encapsulated films preserved meat quality by reducing lipid oxidation and stabilizing meat color. These findings suggest that the incorporation of silver oxide nanocomposites using encapsulation technology not only enhances the mechanical and chemical properties of the films but also enables the development of intelligent and active packaging solutions to extend food shelf life. These advancements have broad applications in food and environmental industries, paving new paths for the development of high‐performance bio‐based films. Overall, this study is significant because it bridges the gap between sustainable biomaterials and advanced food preservation technologies. The findings highlight that plant‐protein‐based films reinforced with encapsulated silver oxide nanocomposites offer a viable, eco‐friendly alternative to synthetic plastic packaging. This approach addresses key challenges in modern food systems by combining biodegradability with antimicrobial and antioxidant functions. The outcomes provide valuable insights for developing next‐generation active and smart food packaging with controlled‐release capabilities, potentially applicable to poultry, seafood, and other perishable products. Moreover, the encapsulation strategy proposed here could guide future innovations aimed at achieving safer, more sustainable, and regulatory‐compliant packaging solutions in the global food industry.

## Author Contributions

**Mohammad Hosein Marhamatizadeh:** supervision, project administration, writing – review and editing. **Sedigheh Yazdanpanah:** project administration, supervision, writing – review and editing, validation. **Mandana Yekta:** writing – original draft, formal analysis, methodology, conceptualization, data curation. **Dornosh Jafarpour:** validation, visualization.

## Conflicts of Interest

The authors declare no conflicts of interest.

## Data Availability

The data that support the findings of this study are available on request from the corresponding author. The data are not publicly available due to privacy or ethical restrictions.
